# 3-Bromo-1-Hydroxy-9,10-Anthraquinone (BHAQ) Inhibits Growth and Migration of the Human Breast Cancer Cell Lines MCF-7 and MDA-MB231

**DOI:** 10.3390/molecules180910367

**Published:** 2013-08-27

**Authors:** Nadiah Abu, M. Nadeem Akhtar, Wan Yong Ho, Swee Keong Yeap, Noorjahan Banu Alitheen

**Affiliations:** 1 Faculty of Biotechnology and Biomolecular Science, Universiti Putra Malaysia, 43400 Serdang, Malaysia; E-Mail: nadyaboo@gmail.com; 2 Bright Sparks Unit, University Malaya, 53500 Kuala Lumpur, Malaysia; 3 Faculty of industrial Sciences & Technology, Universiti Malaysia Pahang, 26300 Lebuhraya Tun Razak, Kuantan Pahang, Malaysia; E-Mail: nadeem409@yahoo.com; 4 The University of Nottingham Malaysia Campus, Jalan Broga, 43500 Semenyih, Selangor Darul Ehsan, Malaysia; E-Mail: WanYong.Ho@nottingham.edu.my; 5 Institute of Bioscience, Universiti Putra Malaysia, 43400 Serdang, Malaysia; E-Mail: skyeap2005@gmail.com

**Keywords:** anthraquinone, BHAQ, cytotoxic, MDA-MB231, MCF-7, migration

## Abstract

Breast cancer is becoming more prominent in women today. As of now, there are no effective treatments in treating metastatic breast cancer. We have tested the cytotoxic and anti-migration effects of BHAQ, a synthesized anthraquinone, on two breast cancer cell lines, MCF-7 and MDA-MB231. Anthraquinones are an interesting class of molecules that display a wide spectrum of biological applications, including anticancer properties. Cellular inhibition was tested through a MTT assay, double acridine orange/propidium iodide staining and FACS cell cycle analysis. Inhibition of migration was tested by the wound healing method, and migration through a Boyden chamber. BHAQ was cytotoxic towards both cell lines in a dose dependent and possibly cell-dependent manner. Additionally, BHAQ also inhibited the migration of the highly metastatic MDA-MB231 cell line.

## 1. Introduction

**Figure 1:**
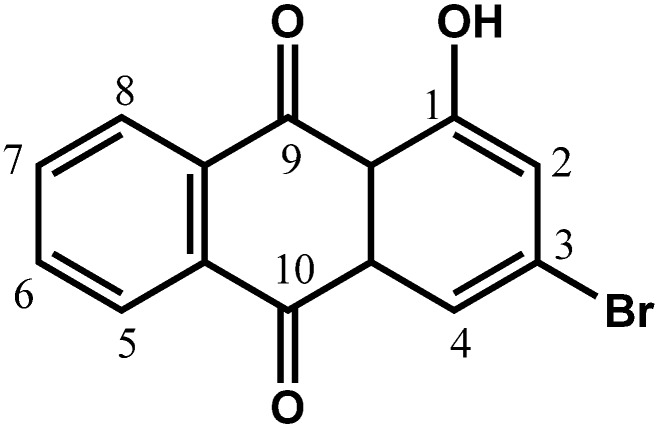
Molecular structure of BHAQ.

Cancer is the number one killer disease in the history of humankind. Among all types of cancers, breast cancer is becoming more prevalent in women [1]. In fact, it is reported that breast cancer is the second leading cause of cancer deaths among women in the United States [2]. Moreover, breast cancer is one of the most highly metastatic cancers. Approximately, 1 in 8 breast cancer patients will develop invasive breast cancer [2]. Metastasis is a process in which primary tumor cells migrate, invade and form secondary tumors at a distant site [3]. When this process takes place, the survival rate of cancer patients decreases significantly, so not only is it important to inhibit tumor growth, it is also as equally vital to prevent the cells from metastasizing. Since Richard Nixon launched a global war to eradicate cancer, research to find the perfect cure has been widely expanding. A variety of chemotherapeutic drugs are being progressively formulated especially in treating metastatic breast cancer. Anthraquinones are among the most active anti-cancer agents present [4,5]. In fact, one of the most famous drugs used to treat breast cancer is doxorubicin, an anthraquinone/anthracycline [4,5]. Nevertheless, despite doxorubicin’s effectiveness in treating breast cancer, it comes with adverse side effects including cardiac toxicity [4,5]. Therefore, in search of a new drug, we attempted to study the cytotoxic and anti-migration effects of chemically synthesized 3-bromo-1-hydroxy-9,10-anthraquinone (BHAQ, Figure 1) on two breast cancer cell lines, MCF-7 and MDA-MB231. 

## 2. Results and Discussion

The MTT assay was performed as a preliminary study of the cytotoxic activity of BHAQ. After treatment with BHAQ, the yellow tetrazolium dye MTT will be reduced by viable cells to a purple formazan substance [6]. This procedure is enabled by the presence of mitochondrial dehydrogenase in viable cells [7]. This assay attempts to measure the IC_50_value, which is the concentration of the drug that can inhibit or kill 50% of the total cells. The results from this assay also imply that this drug affects the mitochondrial potential of these cancer cell lines [8]. Table 1 shows the mean average of IC_50_ values obtained at 48 h post-treatment in MCF-7, MDA-MB231 and MCF-10A. BHAQ had a slightly lower IC_50_ value in MDA-MB231 than MCF-7. BHAQ also exhibited a marginally higher inhibition as compared to tamoxifen in both cell lines. Doxorubicin’s IC_50_, was extremely low as compared to both BHAQ and tamoxifen in both MCF-7 and MDA-MB231. Nevertheless, both doxorubicin and tamoxifen exhibited higher cytotoxicity in the non-cancerous breast cell line, MCF-10A than BHAQ. The selectivity index (SI) of each drug was calculated by obtaining the ratio of IC_50_ in the non-cancerous breast cell line/IC_50_ in the cancerous cell line [9]. The SI value of BHAQ is higher than both tamoxifen and doxorubicin. These results suggest that BHAQ is cytotoxic towards MCF-7 and MDA-MB231cancer cell lines and is more selective than tamoxifen and doxorubicin. 

**Table 1:** The IC_50_ values of BHAQ, tamoxifen and doxorubicin in MDA-MB231, MCF-7 and MCF10A cell lines at 48 h post-treatment. The selectivity index of each drug was also measured, IC_50_ in non-cancerous MCF-10A cell line/IC_50_ in cancerous cell line. All data are expressed as mean ± SD. * *p* < 0.05 compared with corresponding MCF-10A.

Cell Lines	BHAQ	Tamoxifen	Doxorubicin
		IC_50_ (μM)	
MDA-MB231	24.40 ± 1.22	25.84 ± 1.60	0.60 ± 0.36
MCF7	29.40 ± 2.05	24.76 ± 0.80	0.41 ± 0.75
MCF-10A	67.88 ± 3.31 *	26.24 ± 1.21	0.31 ± 0.18
Selectivity Index			
MCF-10A/MDA-MB231	2.78	1.01	0.52
MCF-10A/MCF-7	2.31	1.06	0.76

**Figure 2:**
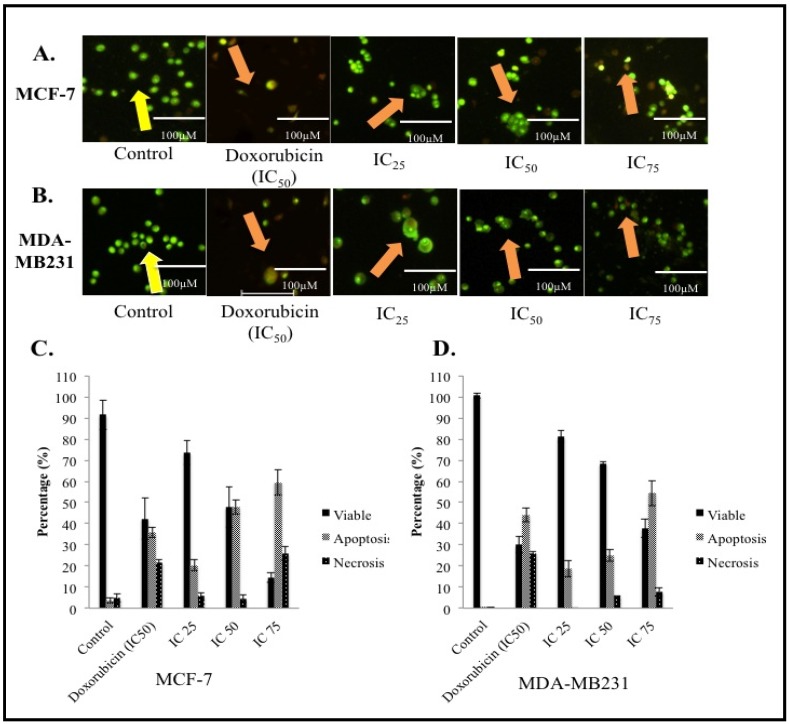
(**A**) Morphological changes in MCF-7 after 48 h of treatment with BHAQ (IC_25_(12.3µM), IC_50_(29.4µM) and IC_75_ (71.2µM)) and IC_50_ of doxorubicin (0.41 µM). (**B**) Morphological changes in MDA-MB231 after 48 h of treatment with BHAQ (IC_25_ (3.3 µM), IC_50_ (24.4 µM) and IC_75_ (92.7 µM)) and IC_50_ of doxorubicin (0.60 ± 0.36 µM). (**C**) Quantification analysis of MCF-7 based on the uptake of acridine orange and propidium iodide in more than 200 cells. (**D**) Quantification analysis of MDA-MB231 based on the uptake of acridine orange and propidium iodide in more than 200 cells. (Yellow arrow: viable; orange arrow: apoptosis). All data are expressed as mean ± SD. * *p* < 0.05 compared with corresponding controls. (Magnification: 100×).

AO/PI staining was carried out to visualize any morphological changes in the cells upon treatment and also to semi-quantitate viable, apoptotic and necrotic cells. Around 90% of viable cells were prominently evident in the control sample of both MCF-7 and MDA-MB231 samples according to Figure 2B and D. This number however, decreased significantly in the treated samples at all concentrations. At 48 h post-treatment for the MCF-7 cell line, the increment of apoptotic cells is dose-dependent. Early apoptotic features were visible even at the lowest concentration of IC_25_ (Figure 2A). Apoptotic cells were categorized based on the presence of classical apoptosis morphological features such as membrane blebbing, protrusion and nuclear condensation [10,11,12]. As seen in Figure 2A and Figure 2C, apoptotic cells can be seen as green, blebbed cells. Similarly, in MDA-MB231 cells, more than 90% of viable cells were present in the control sample and the number decreased in proportion to the dose of BHAQ given. According to Figure 2C apoptotic cells were seen in all treated samples, including in the IC_25_ sample. These results suggest that BHAQ induces apoptosis at all concentrations and the percentage of apoptotic cells is increased as the dose is elevated. Both cell lines were treated with doxorubicin at a concentration of IC_50_ as a positive control. Figure 2A–D show that doxorubicin induces apoptosis in both cell lines. This is evidenced by the presence of apoptotic morphological changes in the cells. Doxorubicin has always been known to induce apoptosis in several cancer cell lines [13]. Drug-induced apoptosis is a major concern in developing new drugs [10,12]. It is a favorable characteristic for anti-cancer agents to induce apoptosis rather than necrosis because necrotic cells may cause inflammation [14]. Additionally the balance between apoptosis and necrosis can be tilted depending on the dose of the drug given. Hence, it is very important to establish a relative connection between the dosage of the drug and the type of cell death it induces. 

**Figure 3:**
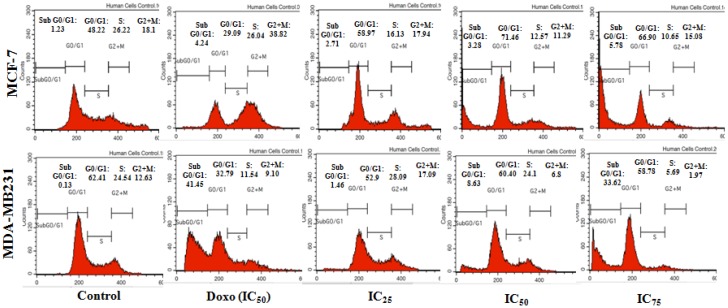
Cell cycle distribution of MCF-7 and MDA-MB231 at 48 h post-treatment when treated with Doxorubicin (IC_50_) and three different concentrations of BHAQ (IC_25_, IC_50_ and IC_75_).

Cancer cells in particular have irregular cell cycle progression profiles due to the mutagenic nature and the presence of growth factors [15,16]. Destruction of the checkpoints is a favourable property in formulating a drug because the cells are more susceptible and sensitive to more damage [12,15]. To determine whether or not BHAQ could affect the cell cycle progression in MDA-MB231 and MCF-7, a cell cycle flow cytometric analysis was done by staining the DNA. According to Figure 3, at 48 h, the percentage of cells in the sub G0/G1 phase in MDA-MB231 samples treated with BHAQ showeda gradual increase as compared to the control. This is in proportion to the concentration of the BHAQ added. This observation was accompanied by a decrease at the S and G2/M phase. For MCF-7 however, there is a significant arrest in the G0/G1 phase at 48 h. Moreover, there is a slight increment in the sub G0/G1 phase as the dose is increased (Figure 3). An arrest at the G0/G1 phase perhaps caused by damage to the DNA [17]. Doxorubicin in MCF-7 induces a G2+M arrest as evidenced by other studies [2,13]. In doxorubicin-MDA-MB231 treated cells, the cell population increased in the subG0/G1 phase instead.

**Figure 4:**
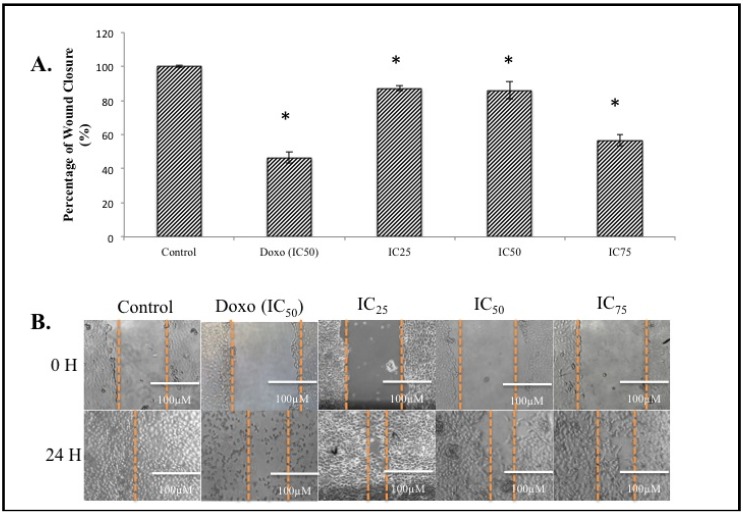
(**A**) The percentage of wound closure of MDA-MB231 cells when treated with BHAQ (IC_25_, IC_50_ and IC_75_) and doxorubicin (IC_50_) after 24 h. (**B**) Figures of the wound at 0 h and 24 h post-treatment. All data are expressed as mean ± SD. * *p* < 0.05 compared with corresponding controls (magnification: 100×).

Metastasis accounts for 90% of cancer related fatalities [18]. Therefore it is imperative for an anti-cancer agent to also offer anti-metastatic abilities. The anti-migration effect of a certain compound is one of the main properties of its inert anti-metastatic activity. To examine the anti-migration properties of BHAQ, a wound-healing assay was performed on the cell line MDA-MB231 since it is highly metastatic compared to MCF-7 [19]. This wound healing assay serves as a preliminary screening assay. It is also a cost-friendly and reliable method [20,21]. A wound is introduced in the middle of the monolayer cells and the ability of the cells to migrate towards the wound was assessed [20,21]. According to Figure 4B, after 24 h, cells filled up the wounded area completely in the control sample. On the contrary, in the BHAQ treated samples, the area of the wounded region differed depending on the dose of BHAQ. As the dose of BHAQ is increased, the size of the wounded area remained bigger. At the end of the assay, samples treated with 25 µM and 50 µM of BHAQ produced an almost similar percentage of wound closure at above 85% of the control (Figure 4A), whereas in the sample treated with 75 µM of BHAQ, BHAQ inhibited the migration of cells down to 56%.This indicates that BHAQ exerts its anti-migration effect at the highest concentration. The rate to which each sample migrated towards the center is dependent on the dose of the drug given. In the positive control expectantly, doxorubicin inhibited the cells from migrating towards the center, the percentage of wound closure is around 50%. This observation correlates with the general use of doxorubicin in treating metastatic breast cancer [4]. Therefore, it can be suggested that the anti-migration effect of BHAQ on MDA-MB231 is dose-dependent. 

**Figure 5:**
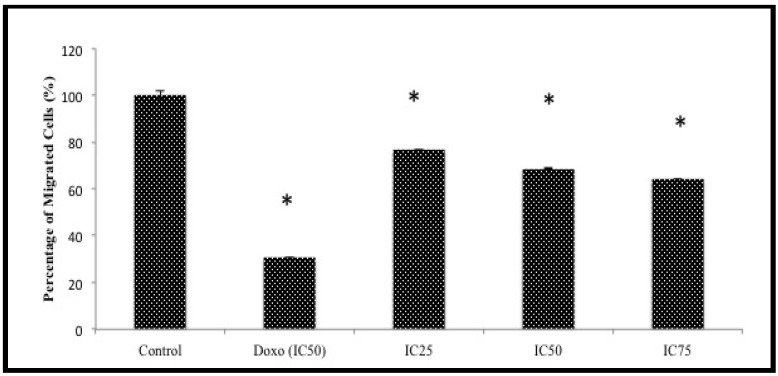
The percentage of migrated cells through the transwell membrane when treated with three different concentrations of BHAQ (IC_25_, IC_50_ and IC_75_) and doxorubicin (IC_50_) after 24 h. All data are expressed as mean ± SD. * *p* < 0.05 compared with corresponding controls.

Another assay was attempted to further screen the anti-migration effects of BHAQ. This assay was designed to observe whether or not the cells could migrate to the other end of a membrane when induced with a chemoattractant [21]. The chart in Figure 5 represents the percentage of MDA-MB231 cells migrating through a Boyden chamber/membrane with a chemoattractant at the bottom of the chamber [22]. The anti-migration effect of BHAQ is subject to gradation in relation to the concentration of BHAQ. The results show that there is a decrease of migrated cells as the dose of BHAQ is increased. At the highest concentration of BHAQ, 75 µM, the percentage of migrated cells was around 60%. Similar to the wound-healing assay, doxorubicin also inhibited the migration of MDA-MB231 cells. These results also imply that BHAQ may inhibit chemotaxis of highly metastatic cancer cells [22].

## 3. Experimental

### 3.1. General

All chemicals used to synthesize BHAQ were obtained from Sigma Chemical Company (St Louis, MO, USA). Dimethylsulfoxide, DMEM, DMEM/F12, insulin, hEGF, RPMI, acridine orange, propidium iodide, RNAse, Triton-X, methanol and crystal violet were all also purchased from Sigma Chemical Company. Tryple E on the other hand was purchased from Invitrogen (Carlsbad, CA, USA). For measurement of absorbance, we used a microtiter plate reader from Bio-Tek Instruments (Bio-Tek Ins, Winooski, VT, USA). For all the FACS analysis, the experiment was done using a FACS Calibur flow cytometer (Becton Dickinson, Franklin Lake, NJ, USA). For imaging analysis, we used a fluorescent microscope by Nikon (Tokyo, Japan). The NMR analysis was performed using a 500 MHz Varian INOVA NMR Spectrometer (Varian Inc, Palo Alto, CA, USA). Mass spectra were acquired using a ThermoFinnigan model LCQ^DECA^ (ThermoFinnigan, San Jose, CA, USA).

### 3.2. Synthesis of BHAQ

3-Bromo-1-hydroxy-9-10-anthraquinone (BHAQ) was synthesized by Friedel Crafts acylation. A mixture of anhydrous aluminium chloride (30 g, 225.0 mmole) and sodium chloride (12 g, 205.0 mmole) were melted at 125–130 °C. Phthalic anhydride (6.7g, 45.0 mmole) and 3-bromophenol (40.5 mmole) were mixed well and added slowly into the molten mixture. The reaction temperature was raised to 165–175 °C and maintained for 45–60 min. After being cooled, the deep red solid product was decomposed by adding a mixture of ice water (250 mL) and conc. hydrochloric acid (250 mL). The crude mixture was dissolved in distilled water and organic layer was extracted with ethyl acetate, washed with brine and dried over anhydrous sodium sulfate. The crude products were purified by flash silica gel column chromatography with elution of the ethyl acetate/hexane as yellow needles. The BHAQ purity was determined by HPLC using a JASCO-HPLC equipped with ChromNAV-software (JASCO Corporation, Tokyo, Japan). HPLC conditions were as follows: column XBridge RP-18 (5-μm particle size, 4.6 × 150 mm i.d.; Waters Corporation, Wexford, Ireland) kept at ambient temperature, injection volume of 20 μL, flow rate at 1 mL/min. and detector wavelength set at 366 nm, mobile phase H_2_O and acetonitrile (30:70), retention time (tR) 9.17 min. The percentage purity of 99.6% was automatically calculated by the peak purity method. Proton NMR data showed no extra peaks in the BHAQ spectra. Yellow crystals, mp 188–189 °C. ^1^H-NMR (acetone-*d_6_*): *δ* H 12.63 (s, 1H, 1-OH), 8.33 (m, 2H, H5, H-8), 7.96 (d, 1H, *J* 2.0, H-4), 7.86 (m, 2H, H6, H-7), 7.52 (d, 1H, *J* 2.0, H-2). *δ* C 188.4 (C9), 181.6 (C10), 163.2 (C1), 135.1 (C7), 134.8 (C6), 134.3 (C14), 133.3 (C12), 133.2 (C11), 131.8 (C13), 127.9 (C8), 127.3 (C5), 127.1 (C2), 123.1 (C4), 115.3 (C3); *m/z* (EI) 304 (66%, [M+ +2]), 302 (63, [M+]), 276 (7), 274 (6), 248 (11), 246 (14), 223 (18), 195 (15), 167 (21), 139 (100), 113 (14), 97 (33), 83 (29),69 (96); *m/z* (%): 301.9558 ([M+], 301.9575 ([M+], 302.1284).

### 3.3. Cell Culture

The cell lines MCF-7, MDA-MB231 and MCF-10a were obtained from the ATCC collection (ATCC, Rockville, MD, USA). MCF-7 was maintained in RPMI supplemented with 10% fetal bovine serum, while MDA-MB231 was maintained in DMEM, also supplemented with 10% FBS. MCF-10A on the other hand, was maintained in DMEM-F12 media supplemented with hydrocortisone (0.5 μg/mL), insulin (10 μg/mL), hEGF (20 ng/mL) and 10% FBS. All the cells were kept in a 37 °C incubator with 5% CO_2_.

### 3.4. MTT Assay

The cells were seeded in a 96-well plate at a concentration of 0.8 × 10^5^ cells/well. The range of treatment for BHAQ, tamoxifen and doxorubicin was at 30 µg/mL followed by 2-fold dilutions. The cell viability was measured at 48 h post-treatment. MTT solution (5 mg/mL, Calbiochem, Darmstadt, Germany) was added at a volume of 20 µL in each well and was incubated for three hours. Then, the solution was removed, and 100 µL of DMSO was added to solubilize the crystals. The plates were then read using a microtiter plate reader at the wavelength of 570 nm (Bio-tek Instruments). Triplicates were carried out for each cell line. The following formula was used to determine the percentage of viable cells:

Percentage of Cell Viability = [OD Sample/OD control] × 100%
(1)

### 3.5. AO/PI Double Staining

Staining of cells with acridine orange and propidium iodide was used to determine the cell viability of MCF-7 and MDA-MB231. The cells were seeded in a 6-well plate at the concentration of 2.3 × 10^5^ cells/well. Both MCF-7 and MDA-MB231 were treated with 3 different concentrations of BHAQ for 48 h. MCF-7 was treated with BHAQ at these concentrations; IC_25_ (12.3 µM), IC_50_ (29.4 µM) and IC_75_ (71.2 µM) and IC_50_ of doxorubicin (0.41 µM). MDA-MB231 on the other hand, was treated with BHAQ at the following concentrations; IC_25_ (3.3 µM), IC_50_ (24.4 µM) and IC_75_ (92.7 µM) and IC_50_ of doxorubicin (0.60 µM). The harvested cells were trypsinized and centrifuged at 2,000 rpm for 5 min. The resulting pellets were resuspended in 100 µL PBS and stained with 10 µg/mL of each dye. Acridine orange is a stain that is permeable to viable cells and can stain the cell’s DNA directly [23]. It emits a green-like fluorescence once it is excited [23]. Propidium iodide on the other hand, is a dye that is impermeable to viable cells [23]. It can bind to DNA only when the cells are dead. It emits a red-orange fluorescence instead [23]. The mixture was viewed under a fluorescent microscope (Nikon). Quantification analysis was done by observing the uptake of acridine orange and propidium iodide in a population of 200 cells on average.

### 3.6. Flow Cytometric Analysis of Cell Cycle Distribution

The cells were seeded in a 6-well plate at the concentration of 2.3 × 10^5^ cells/well. Both MCF-7 and MDA-MB231 were treated with three different concentrations of BHAQ for 48 h. MCF-7 was treated with BHAQ at these concentrations; IC_25_ (12.3 µM), IC_50_ (29.4 µM) and IC_75_ (71.2 µM) and IC_50_ of doxorubicin (0.41 µM). MDA-MB231 on the other hand, was treated with BHAQ at the following concentrations; IC_25_ (3.3 µM), IC_50_ (24.4 µM) and IC_75_ (92.7 µM) and IC_50_ of doxorubicin (0.60 µM). The cells were trypsinized and centrifuged at 2,000 rpm for 5 min. The resulting pellets were fixed in 70% (v/v) ethanol and stored at −20 °C. After a week, the fixed cells were washed with PBS and treated with RNAse and Triton-x, and were then stained with PI. Afterwards, the cells were subjected to flow cytometric analysis using the flow cytometer.

### 3.7. Wound Healing Assay

Monolayer cells of MDA-MB231 were grown to confluency in a 6-well plate. MDA-MB231 was treated with BHAQ at the following concentrations; IC_25_ (3.3 µM), IC_50_ (24.4 µM) and IC_75_ (92.7 µM) and IC_50_ of doxorubicin (0.60 µM). A wound was introduced using a yellow tip pipette in the center of the well. Pictures of the wounded area were captured using an inverted microscope (Nikon) every 3 h, up until 24 h post-treatment.

### 3.8. Transmembrane Migration Assay

MDA-MB 231 cells were serum-starved 24 h prior the assay. The cells were collected and inserted into an 8 µm insert membrane (BD, USA) at a concentration of 3 × 10^5^ cells/well. At the bottom of the membrane, 3T3 conditioned medium was added as a chemoattractant together with different concentrations of BHAQ IC_25_ (3.3 µM), IC_50_ (24.4 µM) and IC_75_ (92.7 µM) and IC_50_ of doxorubicin (0.60 µM). The following day the membrane was fixed with methanol and stained with crystal violet. The dye was then extracted using 30% acetic acid and the concentration was measured at 590 nm. The percentage of migrated cells was measured using the same formula as MTT.

### 3.9. Statistical Analysis

Data was expressed as mean ± standard deviation (S.D.) of at least three independent experiments. Statistical analysis was performed using one-way analysis of variance (ANOVA) followed by Bonferroni’s multiple comparison test. This was accomplished via the GraphPad Prism, V6.0 (GraphPad Software, San Diego, CA, USA). * *p*-value < 0.05 was considered statistically significant.

## 4. Conclusions

Through our results it can be concluded that BHAQ is an interesting molecule that could be a potential anti-cancer agent. It is cytotoxic towards both breast cancer cell lines MDA-MB231 and MCF-7, but is more selective towards the non-cancerous breast cell line MCF-10A compared to tamoxifen and doxorubicin. The morphological changes in treated cells indicate that BHAQ induces apoptosis instead of necrosis, which is a more desirable method of cell death when developing drugs. Furthermore, BHAQ induces a G1 arrest in MCF-7, and this property can be a new target approach in further studies. BHAQ also possesses potential anti-migration properties, which can be further explored to its advantage. Although doxorubicin is more potent than BHAQ, due to doxorubicin’s poor selectivity, BHAQ may be a better compound. Nevertheless, further *in vivo* analysis should be done to assess the antitumor and antimetastatic effects of BHAQ. Overall, BHAQ may be a highly promising candidate for developing a novel anti-cancer drug.
